# Five things to consider before proposing that a circular RNA is a viroid

**DOI:** 10.1371/journal.ppat.1012958

**Published:** 2025-03-07

**Authors:** Jian Wu, David M. Bisaro

**Affiliations:** 1 State Key Laboratory for Managing Biotic and Chemical Threats to the Quality and Safety of Agro-products, Key Laboratory of Biotechnology in Plant Protection of MARA, Key Laboratory of Green Plant Protection of Zhejiang Province, Institute of Plant Virology, Ningbo University, Ningbo, China; 2 Department of Molecular Genetics, Center for Applied Plant Sciences, Center for RNA Biology, and Infectious Diseases Institute, The Ohio State University, Columbus, Ohio, United States of America; Shanghai Center for Plant Stress Biology, CHINA

## Introduction

**Fig 1:**
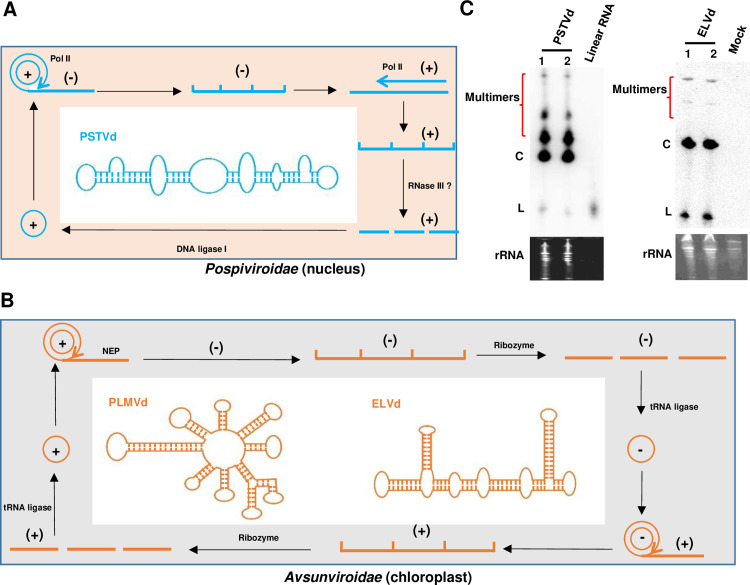
Replication and structural characteristics of known viroids. (**A**) Replication of viroids in the *Pospiviroidae*. Viroids in this family, such as potato spindle tuber viroid (PSTVd), replicate in the nucleus using host enzymes, including DNA-dependent RNA polymerase II, DNA ligase I, and RNase III. Replication occurs via an asymmetric rolling-circle mechanism that produces multimeric RNA intermediates. These are processed and ligated into circular monomers, which adopt a rod-like structure stabilized by extensive intramolecular base pairing. (**B**) Replication of viroids in the *Avsunviroidae*. Avsunviroids such as eggplant latent viroid (ELVd) and peach latent mosaic viroid (PLMVd) replicate within the chloroplast via a symmetric rolling-circle mechanism, utilizing nuclear-encoded chloroplast RNA polymerase for transcription. During replication, hammerhead ribozymes (HHRz) facilitate site-specific self-cleavage of multimeric RNA intermediates. The process relies on tRNA ligase to ligate these cleaved RNA fragments into circular RNA monomers. The mature circular forms of ELVd and PLMVd adopt branched structures stabilized by extensive intramolecular base pairing, ensuring their stability and functionality. (**C**) Identification of viroid RNAs by northern blot. RNA samples extracted from plants infected with PSTVd or ELVd were treated to detect viroid forms. Northern blot analysis using probes complementary to the positive strand of viroids revealed distinct bands corresponding to circular (C) and linear (L) forms, as well as multimeric RNA intermediates generated during rolling-circle replication.

Viroids, small, circular infectious RNAs traditionally associated with plant diseases, have long been a subject of interest in molecular biology [1]. Viroids are classified into two families based on their replication sites and the host factors they utilize. Both families replicate via a rolling-circle mechanism. Members of the *Pospiviroidae*, exemplified by potato spindle tuber viroid, replicate in the nucleus with the aid of host enzymes, including DNA-dependent RNA polymerase II, DNA ligase I, and RNase III (Fig 1A). In contrast, viroids in the *Avsunviroidae*, such as eggplant latent viroid and peach latent mosaic viroid (PLMVd), replicate within the chloroplast. In this process, hammerhead ribozymes (HHRz) take the place of RNase activity, and replication depends on nuclear-encoded chloroplast RNA polymerase and tRNA ligase (Fig 1B) [2].

Initially considered specific to plants, recent discoveries have revealed viroid-like RNAs in diverse ecosystems, including prokaryotic systems, suggesting that these entities may have a much broader host range and ecological relevance than previously understood [3–7]. These viroid-like RNAs are characterized by their circularity and conserved secondary structures, which are typically inferred from computational models without experimental testing for biological properties characteristic of true viroids. In addition, a small circular RNA (circRNA) lacking extensive secondary structure circRNA was recently isolated from colorectal cancer patients, for which the name CRC-associated viroid was proposed [4]. However, classification of circRNA as a true viroid or a viroid-like agent requires more than a circular nature. Several criteria must be met, including an in-depth understanding of replication mechanism, ability to infect hosts and potentially induce disease, and the presence of specific biochemical activities like self-cleavage. In this context, five key criteria based on prior viroid research are outlined here to assist researchers in determining whether a circRNA might qualify as a viroid. These are not intended to limit the discovery of novel functions or characteristics of viroids or viroid-like RNAs, but rather to provide a framework for classification and investigation.

## Lack of protein-coding potential

Although some non-coding RNAs, such as long non-coding RNAs and circRNAs, have been shown to encode small peptides [8–10], efforts to identify proteins or small peptides encoded by viroids have, to date, been unsuccessful [11]. Therefore, it is necessary to analyze the nucleotide sequence of circRNA for open reading frames (ORFs). Bioinformatics tools can be used to predict ORFs and assess the absence or presence of potential protein-coding regions. If putative ORFs are identified, experimental validation, such as LC–MS analysis, should be conducted to detect peptides corresponding to the predicted proteins and confirm their biological relevance [12]. Absence of protein-coding potential would support the classification of the circRNA as a viroid-like entity. Additionally, phylogenetic analysis comparing circRNA sequences with established viroids could reveal evolutionary relationships and provide further evidence for classification as a viroid.

## Presence of functional RNA structures

Viroids are characterized by small, circular, single-stranded RNA genomes, generally ranging from 220 to 450 nucleotides. In addition, they exist as highly structured RNAs with rod-like or branched configurations due to extensive intramolecular base pairing (Fig 1A and 1B) [13–15]. Structural integrity is crucial for stability and enabling resistance to ribonucleases. The compact secondary structure may also limit accessibility to the RISC complex and RNA silencing, contributing to viroid persistence and shaping their evolution [16]. Most importantly, as viroids do not encode protein, replication, and spread in the host relies entirely on secondary and tertiary (3D) structural motifs that are critical for distinct steps in the infection process [16–20]. In contrast, most circRNAs, with the exception of those derived from viruses or specific genes, are formed through back-splicing and tend to exhibit less structured conformations, rendering them more vulnerable to RNA silencing [21,22]. Unlike viroids, circRNAs primarily function in gene regulation, such as miRNA sponging, but their structural flexibility renders them vulnerable to degradation [23]. To confirm that a circRNA is a viroid, it is (of course) essential to verify circularity. This can be accomplished by treatment with RNase R, which selectively degrades nearly all linear RNAs, followed by northern blot analysis. Additionally, circRNAs migrate differently than linear RNAs of similar molecular weight under various gel electrophoresis conditions in both denaturing and non-denaturing gels [24]. RNA sequencing offers a high-throughput approach for detecting circRNAs by identifying unique back-splicing junctions, which are characteristic of circular forms [25]. The use of multiple complementary methods can provide a robust confirmation of RNA circularity. While more challenging, it is also important to investigate potential secondary and tertiary structures for functionality.

## Ability to replicate in the absence of helper virus

Viroids in both the *Pospiviroidae* and *Avsunviroidae* replicate autonomously through an RNA–RNA rolling-circle process driven by host enzymes [26]. During replication, viroids produce multimers of both the plus and minus strands, which can be detected by northern blot. In viroids of both families, the functional circular form (C), which migrates more slowly than the linear form (L) on a PAGE gel, can be readily distinguished using northern blot analysis. Additionally, multimeric forms of viroid RNA can often be detected under optimal conditions (Fig 1C). For a circRNA to be a true viroid rather than a circular viral satellite RNA (virusoid) or some other entity, it must be able to replicate autonomously (by rolling circle or some other mechanism) within host cells, independent of any helper virus [27]. Therefore, the replication potential of novel viroid-like RNAs should be evaluated in potential host cells known to be free of endogenous viruses. While absence of a latent helper virus can be difficult to prove with absolute certainty, approaches similar to those employed by Diener could be employed [27], such as inoculating multiple hosts of the same or related species with purified circRNA, or attempting to enhance its replication by adding extracts from uninoculated hosts to the purified inoculum. Alternatively, deep sequencing and bioinformatics could be used to search for virus-related sequences in host cells.

## Infectivity, transmissibility, and pathogenicity in hosts

Viroids are infectious agents that naturally infect hosts and spread primarily through mechanical transmission, often via contaminated tools or sap transfer [28]. Viroids can also be transmitted through insect vectors, seeds, and pollen; however, these transmission modes typically exhibit low efficiency [29–31]. Most viroids, though not all, are known to cause disease in their hosts. The discovery of circular viroid-like circRNAs in a multitude of hosts outside the plant kingdom (fungi and possibly bacteria and mammals) is a new development that promises to reveal some exciting new biology. However, it is unclear whether these entities, like viroids, are infectious agents. Infectivity, transmissibility and potential pathogenicity must be tested. Methods for testing viroids in plant hosts are well established, and similar methods can be applied, with suitable variation, to other systems. As a first step, evaluating infectivity involves inoculating healthy cells with a circRNA and monitoring replication and possible symptom development over time. The agent should then be re-isolated, characterized, and tested for transmissibility (modified Koch’s Postulates). It is important to acknowledge that not all viroids induce diseases in their respective hosts; thus, pathogenicity should not be regarded as a fundamental criterion for classifying a circRNA as a viroid. Nevertheless, the practical implications of pathogenicity are substantial. In instances where overt symptoms are lacking, molecular and genetic techniques can effectively evaluate host responses at the transcriptomic and/or proteomic levels. Furthermore, longitudinal studies can be implemented to monitor gradual alterations in cell behavior or population dynamics that may result from interactions with viroid-like RNAs.

## Ribozyme activity

Viroids in the *Avsunviroidae* contain catalytically active RNA molecules, typically HHRz, which mediate self-cleavage. During rolling-circle replication, these ribozymes facilitate the site-specific cleavage of long, multimeric RNA strands into monomeric units, a key step in the maturation of viroid genomes [1]. Many circRNAs with predicted ribozyme activity have been identified through metatranscriptomics [3,32]. The demonstration of self-cleavage activity in a novel circRNA can support its classification as a viroid or viroid-like RNA. To assess self-cleavage activity, *in vitro* transcripts, ideally in the form of head-to-tail dimers, can be analyzed using denaturing PAGE. Known viroids with HHRz activity, such as PLMVd, can serve as controls. Self-cleavage is indicated by distinct RNA fragments on the gel, with further validation requiring RACE and sequencing of cleavage products to identify the catalytic site. Sequence and structural analyses can confirm the presence and characteristics of the ribozyme [12,33–35].

## Conclusion

Confirming that a novel circRNA functions as a viroid requires a multi-faceted analysis that includes structural, biochemical, and biological studies. The absence of protein-coding potential, verified through bioinformatics and experimental validation, is one crucial factor distinguishing viroids from other circRNAs. Another is the ability to replicate autonomously in the absence of a helper virus. Additionally, the compact, stable structure of viroids is critical for their function and resilience against host defense mechanisms, and the rolling-circle replication process further sets them apart. Transmission studies, which establish the ability of a circRNA to infect and spread among hosts, are essential for determining its pathogenicity. The presence of ribozyme activity, particularly HHRz, which mediate self-cleavage during replication, provides strong evidence for classification as a viroid. Finally, phylogenetic analysis, comparing the novel circRNA to established viroids, could offer insights into evolutionary relationships that can further substantiate the viroid classification. Together, these integrated methodologies ensure that a viroid-like circRNA is rigorously evaluated, ultimately confirming its identity as a true viroid or a related infectious RNA.
